# How does external reference pricing work in developing countries: evidence from Iran

**DOI:** 10.3389/fphar.2023.1034229

**Published:** 2023-06-20

**Authors:** Fatemeh Babaie, Mohammad Hossein Motevalli, Gholamhossein Mehralian, Farzad Peiravian, Nazila Yousefi

**Affiliations:** ^1^ Department of Pharmacoeconomics and Pharma Management, School of Pharmacy, Shahid Beheshti University of Medical Sciences, Tehran, Iran; ^2^ Nottingham Business School, Nottingham, United Kingdom

**Keywords:** pharmaceutical, reference country, reference pricing, pricing, external reference pricing

## Abstract

**Introduction:** Governments apply different pricing policies to ensure public accessibility, availability, and affordability of medicines. In this way, external reference pricing (ERP) because of its easy implementation is used widely across countries. However, ERP is completely path dependent, and it would both bring pros and cons, related to its implementing strategy which makes understanding of its impact in different countries challenging. In this study, we examine the performance of the ERP approach in Iran as a pricing tool.

**Method:** We conducted a cross-sectional descriptive study. Although Iran officially uses a reference country basket for ERP, in this study, we use different reference countries based on socioeconomic comparability, access to their price data, medicine pricing approaches, and pharmaceutical expenditure to examine the effect of reference countries as well as the method performance. Then, an empirical study was applied to a list of selected samples of medicines in the Iranian market to compare their price with our new reference countries. Then, we discuss the performance of ERP process based on the real prices in the Iranian pharmaceutical market.

**Result:** The prices of 57 medicines, which contain about 69.2% of the imported Iran pharma market in value, were compared with their prices in selected reference countries. It was found that 49.1% of prices were more expensive in at least one of the reference countries, and in 21% of products, the average price in Iran was higher than the average price in reference countries.

**Conclusion:** Achieving efficient and fair pricing of pharmaceuticals between and within countries is still a complex conceptual and policy problem that ERP in short term can handle. ERP cannot be considered a perfect tool for pricing alone, although its effectiveness is acceptable. It is expected that using other pricing methods alongside the ERP will improve patients’ access to medicines. In Iran, we use value base pricing as the main pricing method for every new molecule. Then, we use other methods such as ERP as a complementary method.

## Introduction

A vital aspect of the effectiveness of the healthcare system is access to medicines. Access to medicines is influenced by supply and demand factors, just like other commodities. Patients may find it difficult to receive healthcare services and medicines due to any demand-side constraints, including those caused by distance, opportunity costs, social, cultural, and educational factors (Ensor and Cooper, 2004; Bigdeli et al., 2013). Similar to that, supply constraints like high cost of research and development, long manufacturing processes, and the introduction of new medicines into the pharmaceutical market, on one hand, and the inelastic nature of medicine prices and pharmaceutical market power, on the other hand, lead manufacturing companies to set higher prices on their pharmaceutical products, which eventually affects access (Brekke et al., 2007; Leopold et al., 2012a; Iravani et al., 2020). The World Health Organization (WHO, 2020) has presented four key principles, namely, rational selection and use of essential medicines, affordable prices, sustainable financing, and reliable health supply systems, in order to integrate government and healthcare providers’ efforts to improve access to essential medicines, which is hampered by high pharmaceutical pricing (Equitable, 2004; Alefan et al., 2018). Accordingly, different medicine pricing policies are applied by governments in different countries to ensure proper access to medicines. Governments generally ordain robust policies for controlling the cost of healthcare system services and combat high pharmaceutical prices to ensure fairness in access and affordability (Alefan et al., 2018; Tordrup et al., 2020).

Pricing policies vary among countries which include cost plus, value-based pricing, pooled procurement, reference pricing, and so forth (Tordrup et al., 2020). External reference pricing (ERP) is one of the popular strategies worldwide to control and inform national pharmaceutical prices (Rémuzat et al., 2015; Kanavos et al., 2017a; Iravani et al., 2020). According to the ERP policy, a pharmaceutical company cannot set a price for its products higher than the price accepted by the reference countries (Iravani et al., 2020).

ERP is widely used around the world as a price controller for both patented and non-patented medicines, as well as for setting prices and assisting policymakers with reimbursement decisions (Kanavos et al., 2010; Geng and Saggi, 2017). WHO defines it as “the practice of using the price of a pharmaceutical product in one or several countries to derive a benchmark or reference price to set or negotiate the price of the product in a given country” (World Health Organization, 2020). ERP’s simplicity and capacity to cut costs in the short term via varied implementation strategies have led to its acceptance as a supportive or key criterion of pricing systems in most nations. This highlights the need of exploring the potential applications of ERP in a variety of contexts (Leopold et al., 2012b; Gill et al., 2019; Kanavos et al., 2020).

The selection of reference countries is the first step in implementing an ERP policy. The number of reference countries and the methods used to select them varies per country, from 30 for Poland by including all European Union (EU) and non-EU countries to 3 for Estonia by taking geographical proximity and comparable gross domestic product (GDP) into account (Gill et al., 2019). In general, a country is selected as a reference and added to a reference basket for another country based on certain criteria, such as socioeconomic comparability as measured by GDP, price comparability of the brand medicine, and country of origin (Rémuzat et al., 2015; Kanavos et al., 2017b; Holtorf et al., 2019).

The next step in determining a price involves looking at the minimum or average price in several different countries serving as references. Additionally, the ERP system may take into account the regularity of price changes and the expiration of patent exclusivity rights, both of which tend to favor further price reduction (Verghese et al., 2019; Kanavos et al., 2020). Currency fluctuations are a further factor to consider. Using a fixed exchange rate results in inaccurate price estimates; the ERP formula should use a moving average of the exchange rate to prevent this effect (Gill et al., 2019). It is generally agreed that while ERP may reduce pharmaceutical costs in the short term, it does not match the governor’s expectations for long-term cost containment if it does not adhere to regulatory standards in an appropriate manner (Holtorf et al., 2019).

As a result, despite its benefits such as ease of use and feasibility of implementation, ERP may produce a “spillover effect,” which is a key concern of pharmaceutical innovation enterprises. As a result, a low-cost medicine in one area would migrate to another, discouraging pharmaceutical companies from hastening their production release in other countries to avoid jeopardizing their prices. Pharmaceutical corporations can protect their pricing power in developed countries by doing so (Danzon et al., 2005; Rémuzat et al., 2015; Verghese et al., 2019; Iravani et al., 2020). A second negative consequence of lowering prices followed by a spillover effect is that it may have a negative impact on the accessibility and availability of medicines over time, as pharmaceutical companies’ incentives for R&D and innovation are diminished (Gill et al., 2019).

The main objective of Iranian pharmaceutical policy is making medicines available at affordable prices (Ministry of Health of Iran, 2023). Iranian Food and Drug Administration (IFDA), a sub-branch of the Ministry of Health, manages and regulates the pharmaceutical sector, and supervises medicine manufacturing, distribution, and importation in Iran. The entry of any medicine into the pharmaceutical market of Iran requires a registration process which can take a year or more. For this purpose, the Iran Drug Selection Committee reviews new medicines for safety, efficacy, and cost-effectiveness before adding them to the Iran Drug List (Fardazar et al., 2019; Movahed et al., 2021).

In 2014, sales of both manufactured and imported medicines totaled about 3.87 billion USD, in which manufactured medicines account for 67% and imported medicines account for 33%. This proportion reached 73% for manufactured *versus* 27% for imported medicines in 2016. Between 1992 and 2016, the value of imported pharmaceuticals grew at an average growth rate of 8.5 percent annually, and the value of imported medicines increased from over 206 million dollars in 1992 to almost 1456 million dollars in 2016 (24).

As a result of the government’s increased emphasis on generic substitution as the primary pharmaceutical policy, a substantial portion of medicines are produced domestically in Iran. However, due to the wide range of diseases, the complexity of treatment processes, and the aging population, the government is forced to import a number of medicines to ensure that access to necessary medicines is not denied (Cheraghali, 2005). Iran has generally used ERP as a pricing strategy for imported medicines (Rahimi et al., 2018; FDA, 2023a). As it is widely argued that ERP does not always result in the lowest prices, the purpose of this study was to investigate the effectiveness of the ERP approach as a pricing tool in Iran. To this end, an imported sample of non-local manufactured medicines was compared to the prices set in some countries that are not presently included as a reference country. This helps us comprehend the extent to which the regulatory body has achieved reasonable pricing for imported medicines and whether the ERP tool is reliable or not.

## Methods

**FIGURE 1:**
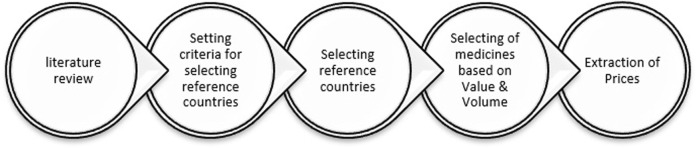
Schematic diagram of the study method.

The present study is a cross-sectional descriptive study. Initially, we searched through scientific articles to identify the primary selection criteria for reference countries. Among the criteria discussed in the literature, such as GDP level, healthcare systems, public health, and wealth situation, we selected socioeconomic situation, medicine pricing strategies, the proportion of pharmaceutical expenditures to GDP, and the status of the pharmaceutical industry (Simoens, 2007; Vogler et al., 2016; Incze et al., 2022). Then, in order to evaluate the current ERP procedure, the prices of selected medicines on the Iranian pharmaceutical market were compared with those of selected reference countries. Currently, the prices of imported pharmaceuticals in Iran are determined by both the country of origin and reference countries, which include Australia, Greece, Spain, Portugal, and Turkey. The utmost price of a brand medicine in Iran should equal its CPT (carrier paid to) minimum price in reference countries. In addition, the cost of imported generic medicines should not exceed 40%–60% of the cost of the same imported brand medicine. After adding the legal taxes paid by importers, the profit margin on imported pharmaceuticals could reach 15%. As the ultimate prices of medicines are determined in Iranian Rials, the price adjustment will depend on the extent to which the currency exchange rate fluctuates over the course of 1 year (FDA, 2023b). The schematic diagram of the study method is shown in Figure 1.

### Selection of reference countries

To conduct the empirical investigation, we first needed to identify some new reference countries that are not currently included in Iran’s regulatory normal procedures.

According to World Bank data, Iran’s GDP *per capita* (PPP) in 2017 was 21011 USD. After reviewing European countries, we discovered that Belarus, Bulgaria, Croatia, Hungary, Romania, Kazakhstan, and Poland have almost the same GDP *per capita* (PPP) as Iran but are not currently used as reference countries for pharmaceutical pricing in Iran (Worldbank, 2021), among which Bulgaria, Hungary, Kazakhstan, Poland, and Romania use the ERP method as a pricing strategy for imported pharmaceutical products (Haiweb, 2023). Given that the proportion of medicines expenditure to GDP in Iran is approximately 1.15%, Hungary and Poland were chosen as our reference countries in this study, with 1.92% and 1.33% of medicines expenditure to GDP, respectively (Oecd, 2021).

Furthermore, we included the United Kingdom (UK) and France as references for several reasons: first, the UK has strict pricing policies imposed by the National Health Service (NHS); second, the UK market is generic dominant (75%), and third, both UK and France are the most referenced for other countries (Oecd, 2011; Thai et al., 2018). Then, we examined the prices of selected medicines in four mentioned countries (Poland, Hungary, France, and UK), and if they were not available in any of the selected countries, we included the price of that particular brand available in the origin country.

### Selection of medicines

Imported medicines approximately account for 30% market share of pharmaceuticals in 2014–2016. Moreover, the value of imported products of pharmaceutical has changed from about 206 million dollars to nearly 1456 million dollars in 1992–2016, respectively (Emamgholipour and Agheli, 2019). Based on the Iranian pharmaceutical statistical report, the top 50 imported medicines in terms of both value and volume were included in the study. The unit price of each medicine was compared according to the same active pharmaceutical ingredient, the same dosage form, and the same medicine brand (Simoens, 2007; FDA, 2023c). After matching and cleaning, the final list of imported medicines contained 87 items.

### Extraction of prices

The consumer price of imported brand medicines was obtained through the National Formulary of Iran (http://irc.fda.gov.ir/nfi) in August and July 2019 (FDA, 2023d).

In order to compare values with available dosage forms in the reference countries, the unit price of each dosage form was calculated. Next, we used the currency exchange rate provided by the Iranian Central Bank to convert the prices of Iranian currency into US dollars (CBI, 2023).

To determine the consumer price of selected medicines in reference countries in August and July 2019, we relied on a website that is utilized in all countries (https://www.cogvio.com/products). We found this website to be sufficiently thorough and trustworthy for extracting prices in the countries of interest.

For imported medicines that are available on the Iranian pharmaceutical market but not in the reference countries, we used the country of origin’s price. However, we were ultimately unable to access the prices of 30 selected medicines, and the prices of the remaining 57 medicines were included in the study.

We used the set price in the country of origin for those imported medicines that are sold on the Iranian pharmaceutical market but not on the markets of reference countries. However, we ultimately did not have access to the prices of 30 chosen medicines, so the prices of the 57 medicines were included in the study.

## Results

Among the total selected imported medicine list, price information for 57 medicines was available in at least one of the four reference countries, accounting for 10.42% of all imported medicines share, with a value accounting for approximately 70% of total imported sales relevant to Iran’s pharmaceutical statistics (FDA, 2023c). France has the lowest average price level among the countries studied, followed by Hungary, the United Kingdom, and Poland, in that order.

**FIGURE 2:**
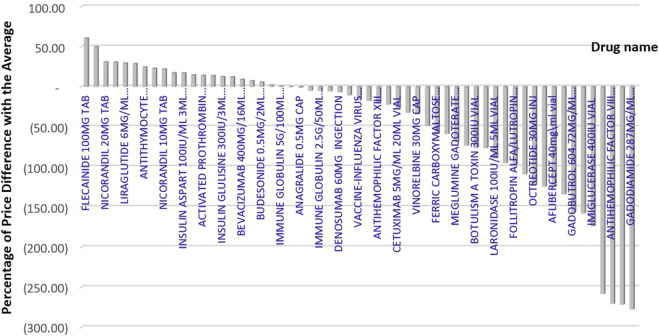
Percentage difference between Iran’s price and the average price of reference countries.

As Figure 2 showing the percentage difference between Iran’s price and the average price of reference countries, the price difference of branded medicines in Iran varies from −278% to +61% compared to the average price of reference countries. We found that, in 36 out of 57 selected medicines, the price of the product available in Iran’s market was lower than the average price in the reference countries. However, 49.1% of selected medicine prices were higher than in at least one of the reference countries.

**FIGURE 3:**
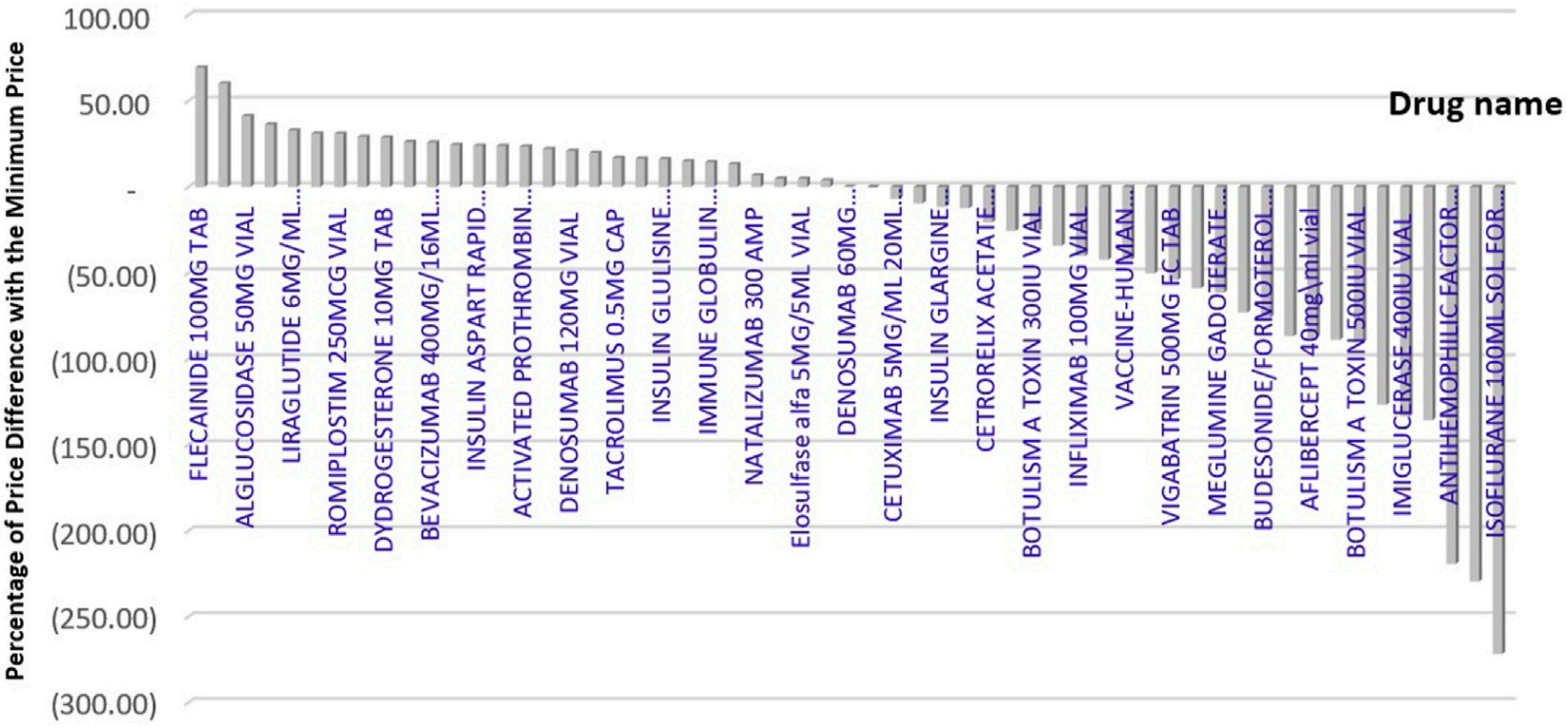
Percentage difference between Iran’s price and the minimum price of reference countries.

Figure 3 displays the price difference between brands in Iran and the minimum price among reference countries, which ranges from −272 to +70 %.

If the price of medicines that are more expensive than the average price in the reference countries is reduced to the average price in the reference countries, we can save approximately 51,798,928 USD while maintaining the same level of access. In addition, if these medicines were provided at the lowest prices available in the reference countries, a total savings of 82,365,722 USD in pharmaceutical costs could be realized.

## Discussion

Given the significant role that medicines play in enhancing public health and saving lives, allocating limited health resources wisely is becoming a challenging issue. It is especially important in the financing of innovative and expensive medicines. This is why practically every country strives to ensure that its patients have access to the most recent pharmaceutical advancements.

For this purpose, governments strive to find an easy-to-use price policy in order to optimize the advantages of their funding resources, in which the external reference policy is one approach that both developed and developing countries frequently adopt (Seiter, 2007; Leopold et al., 2012b).

In the absence of ERP, pharmaceutical companies have a greater incentive to price their medicines affordably so that they are accessible to all (Elek et al., 2017). However, the existence of ERP could threaten pharmaceutical innovations, causing pharmaceutical companies to make price-volume agreements, tighten their international pricing corridor, or even withdraw their product or delay their launches, resulting in inequity in patient access (Csanádi et al., 2018; Incze et al., 2022).

In the conducted study, ERP was able to price about half of the medicines at the minimum price of the newly selected reference countries, despite the fact that half of the prices were higher than the average or minimum of those countries. As in lower-income European Union countries, pharmaceutical expenditures are greater than in higher-income countries (Kaló et al., 2015; Elek et al., 2017). As a result, alternative pricing strategies should be implemented to have the most impact on pricing. For instance, value-based pricing may be appropriate for a number of recently launched medicines. Considering such an approach generally evaluates the clinically added value of a given medicine against the cost of medicines and the country’s economic conditions, or internal reference pricing (IRP) which is appropriate for medicines with similar alternatives. In addition, ERP can be a relatively efficient policy if the following factors for maximizing its benefit are taken into account: policymakers should develop a transparent ERP system with clear definitions, objectives, and usage, and the implementation type of ERP should be linked with health system priorities (Danzon et al., 2005; Gill et al., 2019; Holtorf et al., 2019), a well-considered selection of reference countries in terms of economic or health status similarities (Oecd, 2011; Leopold et al., 2012b; Kanavos et al., 2017b), selective and consistent price revisions to encourage the launch of new products (Alefan et al., 2018), minimizing referrals to low-income countries in order to prevent pharmaceutical launch delays (Kanavos et al., 2017b), and avoid merely referring to the prices of expired patent medicine, while in some countries, the patent is still in effect, using a moving average of the exchange rate to avoid the currency volatility (Thai et al., 2018). Finally implementing the ERP policy in conjunction with other pricing strategies will increase efficiency (Danzon et al., 2005).

In the previous pricing rules in Iran, cost plus was the only approach for domestic production and ERP for imported medicines, but in 2012, due to sanctions against Iran and the problem of currency devaluation (Iran faced a sharp devaluation of the domestic currency against the USD), the supply of affordable medicines in the country decreased. Iran Food and Drug Administration made parallel imports to access medicines at reasonable prices and realized that many of the same imported medicines can be obtained at lower prices, even up to 80% (Yousefi et al., 2019). This issue confirms the achievements of the present article regarding the ineffectiveness of the ERP method. Although in the past few years, the pricing rules of medicines in Iran have been updated, but the pricing method is still one of the challenging issues of medicine regulation in Iran (Yousefi et al., 2019).

Value-based pricing is used to determine the price selling for all new molecules currently; then, complementary pricing strategies such as internal reference pricing, external reference pricing, and cost plus are utilized.

Eventually, since prices fluctuate over the course of a product’s life cycle, medicine pricing should be a dynamic process. Regulatory organizations should actively monitor the cost of medicines in the market, as patent expiration or the introduction of new treatment options may influence prices.

### Study limitations

In conducting this investigation, the following limitations must be mentioned. The result is derived from a sample, and all medicines in the market are not evaluated. In addition, as this is a cross-sectional study, the outcomes may vary for other time periods. Finally, the prices in reference countries were not homogeneous in all cases, and some estimation was made.

## Data Availability

The original contributions presented in the study are included in the article/Supplementary Material; further inquiries can be directed to the corresponding author.
